# Long-Term Bonding Performance of One-Bottle vs. Two-Bottle Bonding Agents to Lithium Disilicate Ceramics

**DOI:** 10.3390/polym16162266

**Published:** 2024-08-09

**Authors:** Masao Irie, Masahiro Okada, Yukinori Maruo, Goro Nishigawa, Takuya Matsumoto

**Affiliations:** 1 Department of Biomaterials, Okayama University Graduate School of Medicine, Dentistry and Pharmaceutical Sciences, 2-5-1 Shikata-cho, Kita-ku, Okayama 700-8525, Japan; tmatsu@md.okayama-u.ac.jp; 2 Department of Dental Biomaterials, Tohoku University Graduate School of Dentistry, 4-1 Seiryo-machi, Aoba-ku, Sendai 980-8575, Japan; masahiro.okada.c2@tohoku.ac.jp; 3 Department of Prosthodontics, Okayama University Hospital, 2-5-1 Shikata-cho, Kita-ku, Okayama 700-8525, Japan; ykmar@md.okayanma-u.ac.jp (Y.M.); goro@s.okayama-u.ac.jp (G.N.)

**Keywords:** shear bond strength, bonding agent, one- vs. two bottles, resin luting materials, lithium disilicate ceramics, durability

## Abstract

The aim of this study was to compare the long-term bonding performance to lithium disilicate (LDS) ceramic between one-bottle and two-bottle bonding agents. Bonding performance was investigated under these LDS pretreatment conditions: with hydrofluoric acid (HF) only, without HF, with a two-bottle bonding agent (Tokuyama Universal Bond II) only. Shear bond strengths between LDS and nine resin cements (both self-adhesive and conventional adhesive types) were measured at three time periods: after one-day water storage (Base), and after 5000 and 20,000 thermocycles (TC 5k and TC 20k respectively). Difference in degradation between one- and two-bottle bonding agents containing the silane coupling agent was compared by high-performance liquid chromatography. With HF pretreatment, bond strengths were not significantly different among the three time periods for each resin cement. Without HF, ESTECEM II and Super-Bond Universal showed significantly higher values than others at TC 5k and TC 20k when treated with the recommended bonding agents, especially at TC 20k. Difference in degradation between one- and two-bottle bonding agents containing the silane coupling agent was compared by high-performance liquid chromatography (HPLC). For both cements, these values at TC 20k were also not significantly different from pretreatment with only Tokuyama Universal Bond II. For LDS, long-term bond durability could be maintained by pretreatment with Tokuyama Universal Bond II instead of the hazardous HF.

## 1. Introduction

As a dental restorative material, lithium disilicate (LDS) outshines traditional metals in many core features. Not only does it have comparable wear resistance, it possesses favorable properties lacking in many dental metals: teeth-mimicking aesthetics and good machinability. The widespread use of LDS is also fuelled by the popular CAD/CAM (computer-aided design and computer-aided manufacture) technology. CAD/CAM is gaining traction in restorative dentistry because it eliminates the reliance on technique-sensitive laboratory procedures and the need to ship restorations. In terms of chairside benefits, CAD/CAM provides a significant leap in patient’s comfort [1,2,3,4,5,6,7,8,9,10,11,12,13,14].

Durable bond strength is critical to the longevity of dental restorations. It is noteworthy that LDS exhibits poor bonding with resin cements. Presently, LDS surfaces are pretreated with hydrofluoric acid (HF) to form micromechanical interlocking force to the end of securing long-term adhesiveness. HF is a weak acid that reacts with and dissolves glass (SiO_2_). However, it is also a dangerous acid that can be easily absorbed into blood through the skin, leading to health hazards such as cardiac arrest [15,16]. Since HF cannot be used intraorally, alternatives such as phosphoric acid, acidulated phosphate fluoride and ammonium hydrogen fluoride have been used as substitutes. However, they fail to achieve HF’s efficacy in bonding durability.

As mentioned above, phosphoric acid is a non-toxic substitute for HF intraorally. However, phosphoric acid could neither create adequate micromechanical retention nor remove silicon oil contamination. As for the effectiveness of phosphoric acid etching on bonding to glass-ceramics, contentious results have been reported. For LDS, bonding effectiveness is pivotally important because compared with other traditional ceramic materials, LDS pales in fracture toughness and defect tolerance. This means that LDS restorations are prone to fracture. Sandblasting is a repair system for intraoral all-ceramic fractures [17], but it risks injuring the surrounding soft tissues and spreading aluminium oxide particles over the operative area [18].

A new type of bonding system categorized as “universal” has been introduced. Universal bonding system is a one-step system that can be applied to enamel and dentin substrates in clinical situations. It can also be applied to bonded restorative materials such as zirconia, metal and silicate-based ceramics without surface pretreatment using a priming agent. This bonding system augurs well for LDS. LDS is a popular glass-ceramic material for single crowns—is compatible with adhesives, self-adhesives and conventional cements [7,8,9]. The introduction of a new universal bonding system thus offers a simplified approach to enhance the bond strength between LDS and resin cements [8].

Many one-bottle bonding agents which contain silane coupling agent are marketed with a prominent emphasis on convenience. The most common silane coupling agent used in dentistry is γ-PTS (gamma-methacryloxy propyl trimethoxy silane) which is diluted in an ethanol-water solution.

To circumvent the deterioration in the adhesion-promoting efficacy of silane coupling agent, two-bottle bonding agents ¾ which separate unhydrolyzed silane from aqueous solution ¾ were introduced [5,6].

Two-bottle bonding agents are reported to provide more durable bond strength than one-bottle bonding agents [17,18,19,20]. In recent years, products containing MPTES [3-(triethoxysilyl) propyl methacrylate], which is more stable than MPS as a silane coupling agent, have also been marketed [21,22].

To obtain more information and insights on the long-term bonding performance of a palette of clinically applied resin cements, this study investigated the adhesive strengths of nine resin cements to LDS with and without HF pretreatment. These nine adhesive and self-adhesive resin cements were selected such that the bonding durability effects of silane coupling agent, one- and two-bottle bonding agents were investigated after one-day water storage (abbreviated as Base), and after 5000 and 20,000 thermocycles (abbreviated as TC 5k and TC 20k respectively). Difference in degradation between one- and two-bottle bonding agents containing the silane coupling agent was compared by high-performance liquid chromatography (HPLC).

The null hypotheses of this study were: (1) two-bottle bonding agent would provide durable bond strength to LDS without HF pretreatment; and (2) bond strength provided by one-bottle bonding agent to LDS would deteriorate over time due to long-term immersion in water, and (3) the usefulness of the HY treatment of Tokuyama Universal Bond II was also examined.

## 2. Materials and Method

### 2.1. Resin Cements and Bonding Agents

Table 1 lists the details of the nine adhesive and self-adhesive resin cements selected for this study. As the focus of this study was to investigate the shear bond strength to LDS with respect to pretreating agent, distinction between two dual-cure resin cements in terms of conventional versus self-adhesive types was deliberately omitted. This range was thus selected to represent the major restorative products used by dentists and to provide a comprehensive, clinically relevant range of values for the parameters under investigation.

Table 2 lists the manufacturer-recommended pretreating agents for the resin cements listed in Table 1. In this study, LDS was also applied with pretreating agent for the self-adhesive type due to the research aims of this study; moreover, adhesion to tooth substrate is different from adhesion to LDS. A single operator (MI) performed all the mixing, handling and bonding procedures according to manufacturers’ recommendations (Table 2). Ten specimens were prepared for each material for evaluation of their mechanical properties at each time period (after one day, and after TC 5k and TC 20k).

For the resin cements, a visible light curing unit (New Light VL-II, GC, Tokyo, Japan; fiber optic tip diameter: 8 mm) was used to irradiate light-activated materials for 20 sec. Using a radiometer (Demetron/Kerr, Danbury, CT, USA), light intensity was checked immediately before the application of each resin cement. During light curing, light intensity was maintained at 450 mW/cm^2^. Since the polymerization of Super-Bond Universal is in self-cure mode, all measurements were made only in self-cure mode. All procedures, except those for shear bond strength measurement, were performed in a thermo-hydrostatic room maintained at a temperature of 23 ± 0.5 °C and relative humidity of 50 ± 2%

### 2.2. Preparation of Lithium Disilicate (LDS; IPS e.max CAD) Surfaces

For each resin cement, 90 custom-made LDS blocks (IPS e.max CAD, Ivoclar Vivadent, Schaan, Liechtenstein; SiO_2_, Li_2_O, K_2_O, P_2_O_5_, ZrO_2_, ZnO, Al_2_O_3_, MagO, coloring oxides; diameter: 5 mm, thickness: 2 mm) were used and each embedded in a slow-setting epoxy resin (Epofix, Struers, Copenhagen, Denmark). Flat LDS surfaces were obtained by grinding with wet silicon carbide paper (#600), then subjected to one of these pretreatments: (1) pretreated with 4.5% hydrofluoric acid (HF; IPS Ceramic Etching Gel, Ivoclar Vivadent, Schaan, Liechtenstein) only for 20 sec; (2) no HF pretreatment; and (3) pretreated with Tokuyama Universal Bond II only.

### 2.3. Shear Bond Strength Measurement

A split Teflon mold with a cylindrical hole (diameter: 3.6 mm, height: 2 mm) was clamped to the prepared LDS surface in a mounting jig. The Teflon mold was filled with each resin cement using a syringe tip (Centrix C-R Syringe System, Centrix, CT, USA). Shear bond strength measurements were performed at three time periods: (i) after one-day storage in distilled water at 37 °C (Base); after (ii) 5000 and (iii) 20,000 thermocycles (thermal stress between 5 and 55 °C; 1 min dwell time), abbreviated as TC 5k and TC 20k respectively.

For each shear bond strength measurement, a shear force was applied using a universal testing machine (Autograph AG-X 20kN, Shimadzu, Kyoto, Japan) at a crosshead speed of 0.5 mm/min. The force was transmitted via a flat (blunt), 1-mm-thick shearing blade at a perpendicular direction to the load. Stress at failure was calculated and recorded as the shear bond strength. Failed specimens were examined under a light microscope at ×4 magnification (SMZ-10, Nikon, Tokyo, Japan) to determine the total number of adhesive failures [14].

### 2.4. High-Performance Liquid Chromatography Analysis

To compare the degradation between one-bottle and two-bottle bonding agents which contained the silane coupling agent.

High-performance liquid chromatography (HPLC) analysis was performed on Scotchbond Universal Plus Adhesive (one-bottle bonding agent) and Tokuyama Universal Bond II (two-bottle bonding agent). In this study, the adhesive of most interest is Tokuyama Universal Bond II, and Scotchbond Universal Plus adhesive, which is widely used worldwide, was used as a control. For these reasons, these two adhesives were used for the measurements. Both bonding agents were described to contain 3-(triethoxysilyl) propyl methacrylate (MPTES) on Safety Data Sheet (SDS).

Measured samples of Scotchbond Universal Plus Adhesive, Tokuyama Universal Bond II and MPTES were each diluted with acetonitrile (CH3CN) containing 0.005 wt.% naphthalene. An LC-20 AD pump (Shimadzu, Kyoto, Japan) was used to deliver the mobile phase to the analytical column at a flow rate of 1.0 mL/min. Chromatographic separation was achieved using a Umisil C18 analytical column (250 mm, 6 mm φ, 5 µm; GL Science, Tokyo, Japan) at 30 °C with CH3CN/H2O. Detection was achieved at a wavelength of 210 nm using an SPD-M20A UV-VIS detector (Shimadzu, Kyoto, Japan). Retention times and absorption wavelengths of HPLC chromatogram peak of MPTES for Scotchbond Universal Plus Adhesive and Tokuyama Universal Bond II were analyzed.

### 2.5. Statistical Analysis

Statistical analysis was performed using the software package, Statistical 9.1 (Statsoft, OK, USA). Analysis of variance (two-way ANOVA) with Tukey-HSD for post-hoc comparison was used to analyze the data obtained for shear bond strength to LDS (*p* < 0.05). Comparisons of the means for shear bond strength to LDS of each resin cement with regard to one- vs. two-bottle bonding agents were done by one-way ANOVA) with *t*-Test (*p* < 0.05). Analyses were conducted using SPSS version 19 (Chicago, IL, USA).

## 3. Results

### 3.1. With and without HF Pretreatment

Table 3 shows the shear bond strength data between LDS and the resin cements with and without HF pretreatment. For each resin cement, adhesive strengths of their HF-treated [HF (+)] specimens were not significantly different among the three time periods (Table 3 and Table 4). Without HF [HF (−)], their adhesive strengths significantly differed over time and became almost zero at TC 20k (*p* = 0.05; Table 3 and Table 4). However, HF (−) specimens of ESTECEM II and Super-Bond Universal showed approximately 20 MPa at TC 20k.

#### 3.1.1. Statistical Comparison among Three Time Periods for Each Resin Cement (Table 3 and Table 4)

For both HF (+) and HF (−) specimens of each resin cement, highest value was yielded at the Base (one-day water storage) time period.

Overall, RelyX Universal Resin Cement showed the greatest values among the six conditions (three time periods, both HF (+) and HF (−) specimens).

With HF pretreatment, Variolink Esthetic DC showed the highest values at TC 5k and TC 20k. Without HF pretreatment, ESTECEM II and Super-Bond Universal showed the highest values at TC 5k and TC 20k. The disparate results shown in Table 3 were statistically classified into many groups (*p* < 0.05) in Table 4.

#### 3.1.2. Statistical Comparison within Each Time Period among Nine Resin Cements (Table 5)

With HF pretreatment, the data were not statistically different within each time period among the resin cements (Table 5).

**Table 5:** Comparison of the means (Tukey-HSD) of shear bond strengths at each time period among the nine resin cements (groups with same superscript letters are not significantly different, *p* > 0.05).

Base		TC 5k		TC 20k	
Pretreating by HF (+)	Pretreating by HF (−)	Pretreating by HF (+)	Pretreating by HF (−)	Pretreating by HF (+)	Pretreating by HF (−)
PANAVIA V5 ^a^	PANAVIA SA Cement Universal ^d^	PANAVIA V5 ^a^	PANAVIA SA Cement Universal ^d^	G-Cem ONE ^a b^	PANAVIA SA Cement Universal ^c^
ResiCem EX ^a^	Nexus Universal Chroma ^d^	Nexus Universal Chroma ^a^	Nexus Universal Chroma ^d,e^	RelyX Universal Resin Cement ^a,b^	Nexus Universal Chroma ^c^
G-Cem ONE ^a^	ResiCem EX ^d^	Super-Bond Universal ^a^	ResiCem EX ^d e^	ResiCem EX ^a,b^	ResiCem EX ^c^
Super-Bond Universal ^a,b^	PANAVIA V5 ^d,e^	ResiCem EX ^a,b^	PANAVIA V5 ^d,e^	PANAVIA V5 ^a,b^	Variolink Esthetic DC ^c^
ESTECEM II ^a,b^	RelyX Universal Resin Cement ^e,f^	PANAVIA SA Cement Universal ^a,b^	G-Cem ONE ^e^	Super-Bond Universal ^a,b^	PANAVIA V5 ^c^
Nexus Universal Chroma ^a,b,c^	G-Cem ONE ^e,f^	G-Cem ONE ^a,b^	RelyX Universal Resin Cement ^f^	ESTECEM II ^a,b^	RelyX Universal Resin Cement ^c,d^
PANAVIA SA Cement Universal ^b,c^	Super-Bond Universal ^f^	ESTECEM II ^b,c^	Variolink Esthetic DC ^f^	Nexus Universal Chroma ^a,b^	G-Cem ONE ^d^
Variolink Esthetic DC ^b,c^	Variolink Esthetic DC ^f^	RelyX Universal Resin Cement ^c^	ESTECEM II ^g^	PANAVIA SA Cement Universal ^a b^	ESTECEM II ^e^
RelyX Universal Resin Cement ^c^	ESTECEM II ^f^	Variolink Esthetic DC ^c^	Super-Bond Universal ^g^	Variolink Esthetic DC ^b^	Super-Bond Universal ^e^

TC 5k: after 5000 thermocycles, TC 20k: after 20,000 thermocycles.

Without HF pretreatment, ESTECEM II and Super-Bond Universal showed significantly better values than the other cements at all the three time periods. Conversely, PANAVIA SA Cement Universal showed significantly lower values than the other cements at all the three time periods.

#### 3.1.3. Statistical Comparison between HF (+) and HF (−) Specimens of Each Resin Cement for Each Time Period (Table 3 and Table 6)

Table 6 shows a total of 27 comparisons (multiple of three time periods for each of the nine resin cements). Each comparison entails a pair of statistical analyses between HF (+) and HF (–) specimens at each time period.

**Table 6:** Comparison of the means (*t*-Test) of shear bond strengths between HF (+) and HF (−) specimens of each resin cement for each time period.

**RelyX Universal Resin Cement/** **Scotchbond Universal Plus Adhesive**			**PANAVIA SA Cement Universal/None**			**PANAVIA V5/** **Clearfil Ceramic Primer Plus**		
**Base**	**TC 5k**	**TC 20k**	**Base**	**TC 5k**	**TC 20k**	**Base**	**TC 5k**	**TC 20k**
S	S	S	S	S	S	S	S	S
**G-Cem ONE/** **G-Cem ONE Adhesive Enhancing Primer**			**ESTECEM II/Tokuyama** **Universal Bond II (A + B)**			**Variolink Esthetic DC/** **Monobond Plus**		
**Base**	**TC 5k**	**TC 20k**	**Base**	**TC 5k**	**TC 20k**	**Base**	**TC 5k**	**TC 20k**
NS	S	S	NS	NS	S	NS	S	S
**ResiCem EX/** **BeautiBond Extreme**			**Nexus Universal Chroma/OptiBond eXTRa Universal**			**Super-Bond Universal/** **M & C Primer A & B**		
**Base**	**TC 5k**	**TC 20k**	**Base**	**TC 5k**	**TC 20k**	**Base**	**TC 5k**	**TC 20k**
S	S	S	S	S	S	NS	NS	S

S: Significant difference (*p* < 0.05), NS: Not significant difference (*p* > 0.05).

On the overall, adhesive strengths of HF (+) specimens were significantly greater than those of HF (−) specimens. However, for ESTECEM II and Super-Bond Universal, there were no significant differences between HF (+) and HF (−) specimens at Base and TC 5k.

### 3.2. Pretreatment with Tokuyama Universal Bond II Only versus Manufacturers’ Recommended Pretreating Agents without HF Pretreatment

Without HF pretreatment, Table 7 presents the shear bond strength values when LDS surfaces were treated with Tokuyama Universal Bond II only versus pretreatment with the respective manufacturer’s recommended pretreating agents. Only values at Base and TC 20k were presented to focus clearly on bonding durability.

#### 3.2.1. Statistical Comparison between Base and TC 20k (Table 8)

In all the comparisons, Base specimens showed statistically better results than those at TC 20k.

**Table 8:** Comparison of the means (*t*-Test) of shear bond strengths between Base and TC 20k for each pretreating agent.

**RelyX Universal Resin Cement**		**PANAVIA SA Cement Universal**		**PANAVIA V5**	
**Tokuyama Universal Bond II (A + B)**	**Scotchbond Universal Adhesive Plus**	**Tokuyama Universal Bond II (A + B)**	**None**	**Tokuyama Universal Bond II (A + B)**	**Clearfil Ceramic Primer Plus**
S	S	S	S	S	S
**G-Cem ONE**		**ESTECEM II**		**Variolink Esthetic DC**	
**Tokuyama Universal Bond II (A + B)**	**G-Cem ONE Adhesive Enhancing Primer**	**Tokuyama Universal Bond II (A + B)**		**Tokuyama Universal Bond II (A + B)**	**Monobond Plus**
S	S	S		S	S
**ResiCem EX**		**Nexus Universal Chroma**		**Super-Bond Universal**	
**Tokuyama Universal Bond II (A + B)**	**BeautiBond Extreme**	**Tokuyama Universal Bond II (A + B)**	**OptiBond eXTRa Universal**	**Tokuyama Universal Bond II (A + B)**	**M&C Primer** **A & B**
S	S	S	S	S	S

TC 20k: after 20,000 thermocycles, S: Significant difference (*p* < 0.05).

#### 3.2.2. Statistical Comparison within Each Time Period among Nine Resin Cements (Table 9a,b)

When treated with their recommended pretreating agents, PANAVIA SA Cement Universal, Nexus Universal Chroma, ResiCem EX and PANAVIA V5 showed significantly lower values than the others at Base. At TC 20k, ESTECEM II and Super-Bond Universal showed significantly better results than the others.

When pretreated with Tokuyama Universal Bond II, significant differences were not statistically pronounced at TC 20k.

**Table 9:** (**a**). Comparison of the means (Tukey-HSD) of shear bond strengths among the nine resin cements at Base and TC 20k and according to pretreating agent (groups with same superscript letters are not significantly different, *p* > 0.05). (**b**). Overview of significant difference groups (Tukey-HSD) at Base and TC 20k according to pretreating agent (groups with same letters are not significantly different, *p* > 0.05).

(**a**)					
**Base (after 1-day)**		**TC 20k**			
**Pretreating by Tokuyama Universal Bond II (A + B)**	**Pretreating by Recommended Bonding Agent**	**Pretreating by Tokuyama Universal Bond II (A + B)**		**Pretreating by Recommended Bonding Agent**	
PANAVIA SA Cement Universal ^a^	PANAVIA SA Cement Universal ^c^	ESTECEM II ^f^		PANAVIA SA Cement Universal ^i^	
Nexus Universal Chroma ^a^	Nexus Universal Chroma ^c^	Nexus Universal Chroma ^f,g^		Nexus Universal Chroma ^i^	
PANAVIA V5 ^a,b^	ResiCem EX ^c^	ResiCem EX ^f,g^		ResiCem EX ^i^	
ESTECEM II ^a,b^	PANAVIA V5 ^c^	Super-Bond Universal ^f,g^		Variolink Esthetic DC ^i^	
RelyX Universal Resin Cement ^a,b^	RelyX Universal Resin Cement ^d^	G-Cem ONE EM ^f,g,h^		PANAVIA V5 ^i^	
G-Cem ONE EM ^a,b^	G-Cem ONE EM ^d,e^	RelyX Universal Resin Cement ^f,g,h^		RelyX Universal Resin Cement ^i^	
Super-Bond Universal ^a,b^	Super-Bond Universal ^d,e^	PANAVIA SA Cement Universal ^f,g,h^		G-Cem ONE EM ^j^	
ResiCem EX ^a,b^	Variolink Esthetic DC ^e^	Variolink Esthetic DC ^g,h^		ESTECEM II ^k^	
Variolink Esthetic DC ^b^	ESTECEM II ^e^	PANAVIA V5 ^h^		Super-Bond Universal ^k^	
(**b**)					
		**Pretreating by Tokuyama Universal Bond II (A + B)**		**Pretreating by Recommended Bonding Agent**	
		**Base** **(after 1-Day)**	**TC 20k**	**Base** **(after 1-Day)**	**TC 20k**
PANAVIA SA Cement Universal		a	f	c	i
Nexus Universal Chroma		a	f,g	c	i
PANAVIA V5		a,b	f,g	c	i
ESTECEM II		a,b	f,g	c	i
RelyX Universal Resin Cement		a,b	f,g,h	d	i
G-Cem ONE EM		a,b	f,g,h	d,e	i
Super-Bond Universal		a,b	f,g,h	d,e	j
ResiCem EX		a,b	g,h	e	k
Variolink Esthetic DC		b	h	e	k

TC 20k: after 20,000 thermocycles.

#### 3.2.3. Statistical Comparison between Two Pretreating Agents at Each Time Period (Table 10)

All conditions showed significantly superior results when treated with Tokuyama Universal Bond II (except Variolink Esthetic DC at Base and Super-Bond Universal at TC 20k).

**Table 10:** Comparison of the means (*t*-Test) of shear bond strengths between two pretreating agents at Base and TC 20k respectively.

**RelyX Universal Resin Cement**		**PANAVIA SA Cement Universal**		**PANAVIA V5**		**G-Cem ONE EM**	
**Base**	**TC 20k**	**Base**	**TC 20k**	**Base**	**TC 20k**	**Base**	**TC 20k**
S	S	S	S	S	S	S	S
**Variolink Esthetic DC**		**ResiCem EX**		**Nexus Universal Chroma**		**Super-Bond Universal**	
**Base**	**TC 20k**	**Base**	**TC 20k**	**Base**	**TC 20k**	**Base**	**TC 20k**
NS	S	S	S	S	S	S	NS

TC 20k: after 20,000 thermocycles, S: Significant difference (*p* < 0.05), NS: Not significant difference (*p* > 0.05).

### 3.3. Chemical Analysis of MPTES by HPLC

**Figure 1:**
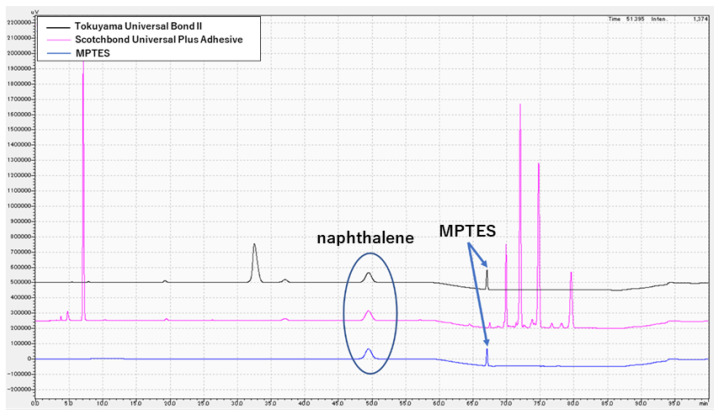
High-performance liquid chromatography (HPLC) results of Tokuyama Universal Bond II (black), Scotchbond Universal Plus Adhesive (pink) and 3-(triethoxysilyl)propyl methacrylate (MPTES) (blue).

**Figure 2:**
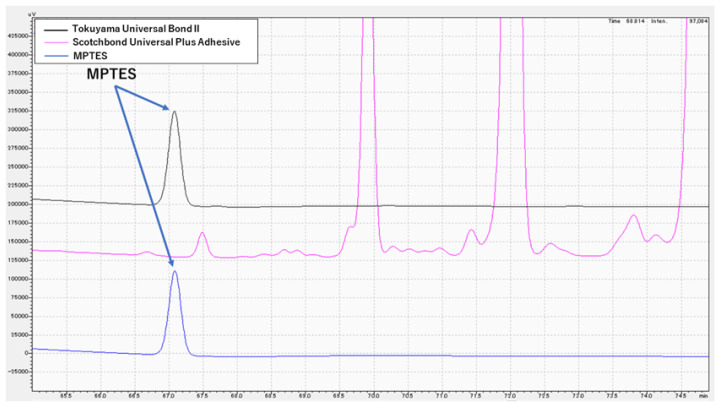
HPLC chromatogram expanded from 65 to 75 min. Tokuyama Universal Bond II (black), Scotchbond Universal Plus Adhesive (pink) and MPTES (blue).

**Figure 3:**
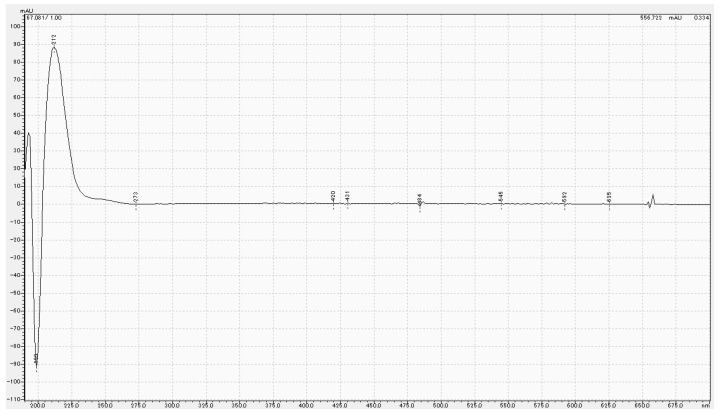
Absorption wavelength of peak at retention time of 67.2 min for Tokuyama Universal Bond II.

**Figure 4:**
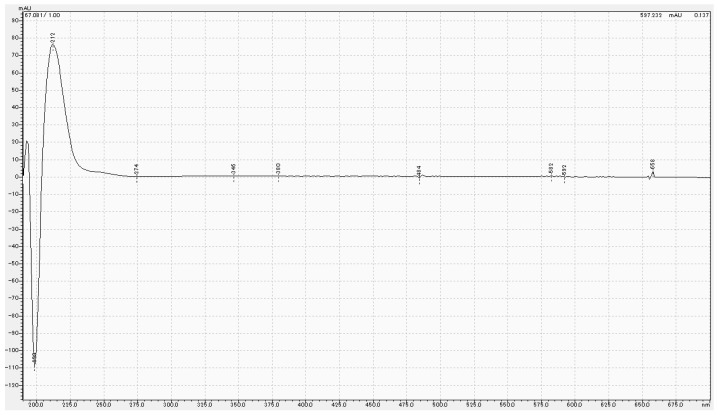
Absorption wavelength of peak at retention time of 67.2 min for MPTES.

HPLC results for Tokuyama Universal Bond II, Scotchbond Universal Plus Adhesive and MPTES are shown in Figure 1 and Figure 2. For Tokuyama Universal Bond II, a peak was identified at the same retention time (67.2 min) as MPTES. Figure 3 shows the absorption wavelength of the peak at retention time 67.2 min for Tokuyama Universal Bond II, whereas that of MPTES is shown in Figure 4. The corresponding peaks showed similar absorption wavelengths. Based on these results, it was confirmed that MPTES was contained in the Tokuyama Universal Bond II product as stated in the SDS.

## 4. Discussion

This study investigated the long-term bonding performance of different pretreatment methods by comparing shear bond strengths between nine clinically applied resin cements and LDS over three time periods: after one-day water storage (Base), TC 5k and TC 20k. The pretreatment methods investigated were: (i) with or without HF; and (ii) one-bottle versus two-bottle bonding agents which contained silane coupling agent.

In this study, thermocycling tests of 20,000 thermocycles were carried out because they aptly represent the conditions in the oral cavity [14]. According to the literature [23], provisional estimate of approximately 10,000 cycles per year was suggested. Therefore, we consider the thermocycles 20,000 times to be a two year in vivo condition.

### 4.1. HF (+) versus HF (−)

When LDS was pretreated with HF, porous concavo-convex surface and micro retentions were produced due to HF selectively dissolving the glassy matrix. Roughness is a vital surface property of restorative materials, influencing the substances’ abrasiveness and mechanical retention despite stresses from the external environment. Surface roughness is not the only determinant of material adhesion; it is also influenced by other characteristics, such as porosity, residual microstructural tension, composition, and mass defects [6].

HF pretreatment created a rough surface which provided micromechanical retention for the resin cement. It also produced microporosity which helped to facilitate resin penetration and significantly improve bond strength [3,17,18,19]. Microporosity also resulted in appreciably larger surface area for interaction with silane to increase wettability.

Increase in wettability led to increase in surface energy. Therefore, HF improved micromechanical interlocking by increasing surface roughness, and it also enhanced chemical bonding with resin cement due to increase in surface energy on silane-treated LDS. Results of this study showed that HF pretreatment followed by silane application resulted in the highest bond strength values. Moreover, the most favourable surface treatment for LDS was pretreatment with HF followed by application of silane coupling agent in a two-bottle adhesive.

Based on the above reasons and the results obtained in this study, it is suggested that HF (+) protocol produced more durable bonding than HF (−) protocol.

### 4.2. One-Bottle versus Two-Bottle

Silane coupling agent application is known to improve the bond strength to LDS and silica-based ceramics. It was reported that chemical bonds between LDS and resin composite luting materials could be achieved by the silanol group of silane molecules reacting with silica on the ceramic surface.

**Figure 5:**
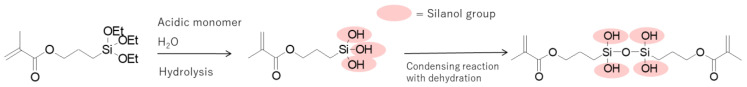
Hydrolysis and condensation reaction mechanism of silane coupling agent (MPTES).

In this study, Tokuyama Universal Bond II and M&C Primer (which were two-bottle bonding agents) provided significantly better long-term durability (TC 20k) to LDS than one-bottle bonding agents. In two-bottle pretreating agents, hydrolysis of the silane coupling agent could occur only when the two components were mixed during use, resulting in the formation of silanol groups (Figure 5). Consequently, the number of silanol groups that could condense and react with LDS (Figure 5) was higher than that yielded by one-bottle pretreating agents. In other words, two-bottle bonding agents provided a higher number of chemical bonds to LDS than one-bottle bonding agents, thereby averting a significant decrease in their bond strengths during durability tests.

Although all LDS surfaces in this study were treated with a silane coupling agent, bond strength results were observed to be material- and time-dependent [16,18,19]. Without HF pretreatment, bond strength results were close to zero at TC 20k, except when pretreated with two-bottle bonding agents. Therefore, results of this study supported the hypothesis that two-bottle bonding agent could provide durable bond strength to LDS without HF pretreatment.

### 4.3. Analysis of MPTES by HPLC

After production, MPTES in Scotchbond Universal Plus Adhesive is expected to decompose because the presence of MPTES, phosphate monomer and water in the same bottle would promote hydrolysis and dehydration condensation of MPTES.

As shown in Table 2, Scotchbond Universal Plus Adhesive contained phosphate monomer, water and MPTES in one bottle. As shown in Figure 5, coexistence of these three components resulted in the hydrolysis of MPTES. Silanol groups were generated, and dehydration condensation between silanol groups led to the oligomerisation of MPTES. With all these reactions occurring in the single bottle, it is suggested that MPTES was thus depleted from the composition, although this was not confirmed by HPLC in this study.

On the other hand, Tokuyama Universal Bond II contained MPTES and water in Liquid B and phosphate monomer in Liquid A (Table 2). As MPTES and water were separated from the phosphate monomer, the hydrolysis of MPTES was unlikely to occur. Hence, the presence of undecomposed MPTES was clearly confirmed by HPLC in this study.

Adhesion to LDS is achieved by condensation reactions between silanol groups in silane coupling agent and silanol groups on LDS surface to form chemical bonds.

In one-bottle pretreating agents, hydrolysis and dehydration condensation of the silane coupling agent occurred within the product, thereby reducing the number of silanol groups in the silane coupling agent. Consequently, the number of silanol groups that could condense and react with LDS was reduced. Chemical bonds formed by the silane coupling agent to LDS became inadequate, such that adhesive strength deteriorated and decreased during durability tests.

In two-bottle pretreating agents, hydrolysis of the silane coupling agent within the product was unlikely to occur before the two bottles were mixed. When silanol groups were formed by hydrolysis of the silane coupling agent during use (that is, when the two bottles were mixed), the number of silanol groups that could condense and react with LDS was higher than that rendered by one-bottle pretreating agents. With adequate chemical bonding to LDS in this case, adhesive strength was not significantly decreased during durability tests.

Therefore, results of this study supported the hypothesis that bond strength provided by one-bottle bonding agent to LDS deteriorated over time due to long-term immersion in water. Deterioration stemmed from inadequate chemical bonding, which in turn stemmed from the hydrolysis of MPTES within the product over time.

### 4.4. Applicability of Tokuyama Universal Bond II as a Pretreating Agent

In this study, when Tokuyama Universal Bond II was used with both adhesive and self-adhesive resin cements, good bond strength results with LDS were obtained [Table 7 and Table 9a]. In other words, a two-bottle bonding agent was useful in attaining durable bond strength between LDS and resin cement.

In addition to the effect of the new functional monomer, New 3D-SR monomer (Table 2), the bonding efficacy of Tokuyama Universal Bond II could be attributed to storage stability at room temperature and the improbability of MPTES hydrolysis and depletion in a two-bottle composition [14,20,22].

For intraoral applications, results of this study showed that Tokuyama Universal Bond II is a viable pretreatment substitute for HF. Beyond the present manufacturer-recommended use, its future application could be expanded to be a general-purpose pretreating agent for bonding between resin cements and LDS

### 4.5. Limitation

Limitations of the present study are inherent to the in vitro design, where only controlled variables were considered. Intraoral temperature changes might influence the long-term outcome of indirect restorations since the different materials employed in this study presented higher thermal contraction/expansion coefficients than teeth [14].

## 5. Conclusions

(i) With HF pretreatment, the shear bond strengths of each resin cement were not significantly different among the three time periods (after one-day water storage, TC 5k and TC 20k). Without HF pretreatment, Tokuyama Universal Bond II and Super-Bond Universal showed the highest values at TC 5k and TC 20k, which were significantly greater than the other resin cements.
(ii) At TC 20k, Tokuyama Universal Bond II and Super-Bond Universal showed significantly better results than others when treated with the recommended bonding agents without HF pretreatment. **These results were not significantly different from the bond strength values at TC 20k when pretreatment was done with Tokuyama Universal Bond II only without HF.**

## Figures and Tables

**Table 1:** Adhesive and self-adhesive resin cements used in this study.

Product	Composition	Manufacturer	Batch No.
RelyX Universal Resin Cement	Surface treated glass powder filler, Phosphate ester monomer, TEGDMA, DiurethaneDimethacrylate, Silica filler, Initiator, Titanium Dioxide	3M, Seefeld, Germany	8697260
PANAVIA SA Cement Universal (Automix)	Paste A: MDP, Bis-GMA, TEGDMA, HEMA, Silanated barium glass filler, Silanated colloidal silica, dl-Camphorquinone, Peroxide, Catalysts, Pigments Paste B: Hydrophobic aromatic dimethacrylate, Silane coupling agent, Silanated barium glass filler, Aluminum oxide filler, Surface treated sodium fluoride (Less than 1%), dl-Camphorquinone, Accelerators, Pigments, Filler content: 40 vol.%, 62 wt.%	Kuraray Noritake Dental, Tainai, Niigata, Japan	RRO194
PANAVIA V5	Paste A: Bis-GMA, TEGDMA, Hydrophobic aromatic dimethacrylate, Hydrophilic aliphatic dimethacrylate, Initiators, Accelerators, Silanated barium glass filler, Silanated fluoroalminosilicate glass filler, Colloidal silica Paste B: Bis-GMA, Hydrophobic aromatic dimethacrylate, Hydrophilic aliphatic dimethacrylate, Silanated barium glass filler, Silanated alminium oxide filler, Accelerators, dl-Camphorquinone, Pigments, Filler content: 38 vol %, 61 wt.%	Kuraray Noritake Dental, Tainai, Niigata, Japan	4EO236
G-Cem ONE EM	Fluoro-alumino-silicate-glass, Urethanedimethacrylate, Dimethacrylate, Phosphoric ester monomer, Silicone dioxide, Initiator, 54~67 wt.%	GC, Hasunuma, Itabashi, Tokyo, Japan	2302201
ESTECEM II	Paste A: Bis-GMA, TEGDMA, Bis-MPEPP, Silica-Zirconia Filler Paste B: Bis-GMA, TEGDMA, Bis-MPEPP, Silica-Zirconia Filler, Camphorquinone, Peroxide, Filler content: 74 wt.%	Tokuyama Dental, Tokyo, Japan	078001
Variolink Esthetic DC	Monomer matrix: urethane dimethacrylate and further methacrylate monomers, Inorganic filler: mixed oxides, ytterbium trifluoride Additional contents: initiators, stabilizers, pigments (<1%) Total content of inorganic filler: approx. 65 wt.%	Ivoclar Vivadent AG, Schaan, Liechtenstein	ZO47JD
ResiCem EX	Paste A: S-PRG filler, Bis-GMA, Silicate glass, Initiators, Others Paste B: S-PRG filler, Bis-GMA, Silicate glass, Initiators, Others Filler content: 65 wt.%, 45 vol.%	Shofu, Kyoto, Japan	062209
Nexus Universal Chroma	TEGDMA, Urethane dimethacrylate, Bisphenol A diglycidylmethacrylate (Bis-GMA), GPDM, photoinitiator, redox initiator, bariumaluminosilicate glass filler, ytterbium fluoride, silica, Filler content: 43 vol.%, 68 wt.%	Kerr, Orange CA, USA	8764312
Super-Bond Universal	PMMA, 4-META, MMA, TBB, Self-cure type (Bulk-mix technique)	SUN MEDICAL, Moriyama, Japan	Universal Polymer: EW1, Catalyst V: FW12

TEGDMA: Triethyleneglycol dimethacrylate, Bis-GMA: Bisphenol A diglycidylmethacrylate, MDP: 10-Methacryloyloxydecyl dihydrogen phosphate, HEMA: 2-Hydroxymethacrylate PMMA: poly(methyl methacrylate), 4-META: 4-methacryloxyethyl trimellitate anhydride, MMA: methyl methacrylate, TBB: Tri-n-butylborane.

**Table 2:** Pretreating agents used in this study.

Adhesive	Batch No.	Composition	Manufacturer	Surface Treatment of IPS e.max
Scotchbond Universal Plus Adhesive	7836013	Brominated dimethacrylate, HEMA, Silane Treated Silica, Vitrabond Copolymer, MDP, Initiators, MPTES, Ethanol, Water	3M, Seefeld, Germany	Scotchbond Universal Plus Adhesive (20 s)—air (5 s)
CLEAFIL CERAMIC PRIMER PLUS	3L0094	3-Methacryloxypropyl trimethoxysilane, MDP, Ethanol	Kuraray Noritake Dental, Tainai, Niigata, Japan	CLEAFIL CERAMIC PRIMER PLUS (1–2 s)—air (5 s)
G-Cem One Multi Primer	2104221	Vinyl silane, Phosphate ester monomer, Thiophosphate ester monomer, Methacrylic ester, Ethanol	GC, Hasunuma, Itabashi, Tokyo, Japan	G-Cem One Multi Primer (10 s)—air (5 s)
Tokuyama Universal Bond II (A + B)	Bond A: 019 Bond B:514	**Liquid A:** Phosphoric acid monomer (New 3D-SR monomer), MTU-6, HEMA, Bis-GMA, TEGDMA, Acetone, Others. **Liquid B:** γ-MPTES, Borate, Peroxide, Acetone, Ethanol, Water, Others	Tokuyama Dental, Tokyo, Japan	Tokuyama Universal Bond II Mix (Liquid A + Liquid B, 1–2 s)—air (5 s)
Monobond Plus	ZO1LG8	Phosphoric acid monomer, Silane methacylate, Ethanol	Ivoclar Vivadent AG, Schaan, Liechtenstein	Monobond Plus (60 s)—air
BeautiBond Xtreme	042347	Acetone, Water, Bis-GMA, TEGDMA, Phosphoric ester monomer, Silane coupling agent, Initiator, Others	Shofu, Kyoto, Japan	BeautiBond Xtreme (20 s)—air
OptiBond eXTRa Universal	Primer: 8199022 Adhesive: 8181793	HEMA, dimethacrylate monomers, tri-functional methacrylate monomer, Ethanol, Photo-initiator, Bariumaluminosilicate filler, Silica, Sodium hexafluorosilicate	Kerr, Orange CA, USA	OptiBond eXTRa Adhesive (15 s)—air (5 s)—LED light (5 s)
M&C Primer A & B	Liquid A: EW1, Liquid B: ES1	M&C Primer A: MDP, VTD, MMA, Acetone M&C Primer B: γ -MPTS, MMA	SUN MEDICAL, Moriyama, Japan	Mix (Liquid A + Liquid B,1–2 s)—air (5 s)

2-HEMA:Hydroxyethylmethacrylate, MDP: 10-methacryloyloxydecyl dihydrogen phos-phate, Bis-GMA: Bisphenol A diglycidylmethacrylate, 4-MET: 4-methacryloxyethyl trimellitic acid, MTU-6: 6-methacryloxyhexyl 2-thiouracil-5-carboxylate, γ-MPTES: 3-(triethoxysilyl) propyl methacrylate, VTD: 6-(4-vinylbenzyl-n-propyl) amino-1,3,5-triazine-2,4-dithione, MMA: methyl methacrylate, γ -MPTS: 3-methacryloxypropyl trimethoxy silane.

**Table 3:** Shear bond strengths between e.max and resin cements with and without HF pretreatment (MPa, mean (standard deviation), Adhesive failures).

Resin Cement/Pretreating Agent	Pretreating by HF	Time		
		Base (1-Day)	TC 5k	TC 20k
RelyX Universal Resin Cement/ Scotchbond Universal Plus Adhesive	(+)	43.4 (5.1, 0)	41.6 (6.2, 0)	29.3 (3.3, 0)
	(−)	30.1 (8.2, 0)	17.0 (4.7, 0)	1.7 (0.9, 0)
PANAVIA SA Cement Universal/None	(+)	38.3 (3.7, 0)	32.6 (5.4, 0)	33.7 (6.6, 0)
	(−)	14.3 (2.6, 0)	1.9 (0.8, 2)	0.9 (0.3, 5)
PANAVIA V5/ Clearfil Ceramic Primer Plus	(+)	30.9 (4.0, 0)	28.3 (2.4, 0)	30.8 (3.7, 0)
	(−)	22.1 (6.2, 0)	5.4 (2.1, 0)	1.5 (0.6, 0)
G-Cem ONE EM /G-Multi Primer	(+)	31.2 (4.6, 1)	34.1 (7.7, 0)	26.2 (3.4, 0)
	(−)	30.4 (7.1, 0)	8.5 (2.4, 0)	7.3 (2.1, 0)
ESTECEM II/ Tokuyama Universal Bond II (A+B)	(+)	33.3 (6.0, 0)	37.9 (4.1, 0)	31.1 (4.9, 0)
	(−)	38.3 (8.2, 0)	27.5 (3.6, 0)	20.5 (4.4, 0)
Variolink Esthetic DC/ Monobond Plus	(+)	41.6 (3.7, 0)	41.7 (5.1, 0)	35.8 (5.5, 0)
	(−)	38.1 (5.8, 0)	21.0 (3.3, 0)	1.3 (0.6, 0)
ResiCem EX/ BeautiBond Extreme	(+)	31.2 (3.3, 0)	31.7 (2.8, 0)	30.5 (3.3, 0)
	(−)	19.4 (3.9, 0)	3.8 (1.4, 2)	1.1 (0.5, 0)
Nexus Universal Chroma/ OptiBond eXTRa Universal	(+)	37.1 (6.1, 0)	29.9 (2.6, 0)	31.8 (4.6, 0)
	(−)	18.0 (4.8, 0)	2.5 (1.5, 0)	0.9 (0.3, 6)
Super-Bond Universal/M&C Primer A & B	(+)	33.2 (3.8, 0)	31.2 (5.5, 0)	30.9 (1.6, 0)
	(−)	31.7 (4.1, 0)	28.2 (5.1, 0)	22.5 (5.2, 0)

HF: 4.5% hydrofluoric acid, TC 5k: after 5000 thermocycles, TC 20k: after 20,000 thermocycles, n = 10, Adh: Number of adhesive failure modes after failure [21].

**Table 4:** Comparison of the means (Tukey-HSD) of shear bond strengths of each resin cement among three time periods (groups with same superscript letters are not significantly different, *p* > 0.05).

**RelyX Universal Resin Cement/Scotchbond Universal Plus Adhesive**		**PANAVIA SA Cement Universal/None**		**PANAVIA V5/Clearfil Ceramic Primer Plus**	
**Pretreating by HF (+)**	**Pretreating by HF (−)**	**Pretreating by HF (+)**	**Pretreating by HF (−)**	**Pretreating by HF (+)**	**Pretreating by HF (−)**
Base ^a^	Base ^c^	Base ^a^	Base ^b^	Base ^a^	Base ^b^
TC 5k ^a^	TC 5k ^d^	TC 20k ^a^	TC 5k ^c^	TC 20k ^a^	TC 5k ^c^
TC 20k ^b^	TC 20k ^e^	TC 5k ^a^	TC 20k ^c^	TC 5k ^a^	TC 20k ^d^
**G-Cem ONE/G-Cem ONE Adhesive Enhancing Primer**		**ESTECEM II/Tokuyama Universal Bond II (A + B)**		**Variolink Esthetic DC/Monobond Plus**	
**Pretreating by HF (+)**	**Pretreating by HF (−)**	**Pretreating by HF (+)**	**Pretreating by HF (−)**	**Pretreating by HF (+)**	**Pretreating by HF (−)**
TC 5k ^a^	Base ^b^	TC 5k ^a^	Base ^a^	TC 5k ^a^	Base ^b^
Base ^a^	TC 5k ^c^	Base ^a^	TC 5k ^b^	Base ^a^	TC 5k ^c^
TC 20k ^a^	TC 20k ^c^	TC 20k ^a^	TC 20k ^c^	TC 20k ^a^	TC 20k ^d^
**ResiCem EX/BeautiBond Xtreme**		**Nexus Universal Chroma/OptiBond eXTRa Universal**		**Super-Bond Universal/M & C Primer A & B**	
**Pretreating by HF (+)**	**Pretreating by HF (−)**	**Pretreating by HF (+)**	**Pretreating by HF (−)**	**Pretreating by HF (+)**	**Pretreating by HF (−)**
TC 5k ^a^	Base ^b^	Base ^a^	Base ^b^	Base ^a^	Base ^b^
Base ^a^	TC 5k ^c^	TC 20k ^a^	TC 5k ^c^	TC 5k ^a^	TC 5k ^c^
TC 20k ^a^	TC 20k ^c^	TC 5k ^a^	TC 20k ^c^	TC 20k ^a^	TC 20k ^d^

**Table 7:** Shear bond strengths between e.max and resin cements without HF pretreatment (MPa, mean (standard deviation), Adhesive failures).

Resin Cement	Pretreating Agent	Time	
		Base (1-Day)	TC 20k
RelyX Universal Resin Cement	Tokuyama Universal Bond II (A + B)	40.1 (5.5, 0)	24.7 (2.1, 0)
	Scotchbond universal Adhesive Plus	30.1 (8.2, 0)	1.7 (0.9, 0)
PANAVIA SA Cement Universal/None	Tokuyama Universal Bond II (A + B)	34.8 (4.9, 0)	25.0 (2.8, 0)
	None	14.3 (2.6, 0)	0.9 (0.3, 5)
PANAVIA V5	Tokuyama Universal Bond II (A + B)	37.2 (4.4, 0)	29.0 (4.0, 0)
	Clearfil Ceramic Primer Plus	22.1 (6.2, 0)	1.5 (0.6, 0)
G-Cem ONE EM	Tokuyama Universal Bond II (A + B)	40.5 (6.5, 1)	24.6 (2.0, 0)
	G-Multi Primer	30.4 (7.1, 0)	7.3 (2.1, 0)
ESTECEM II/Tokuyama Universal Bond II	Tokuyama Universal Bond II (A + B)	38.3 (8.2, 0)	20.5 (4.4, 0)
Variolink Esthetic DC /Monobond Plus	Tokuyama Universal Bond II (A + B)	43.1 (4.0, 0)	25.1 (4.2, 0)
	Monobond Plus	38.1 (5.8, 0)	1.3 (0.6, 0)
ResiCem EX /BeautiBond Xtreme	Tokuyama Universal Bond II (A + B)	42.6 (6.9, 0)	23.7 (3.6, 0)
	BeautiBond Extreme	19.4 (3.9, 0)	1.1 (0.5, 0)
Nexus Universal Chroma	Tokuyama Universal Bond II (A + B)	34.9 (4.3, 0)	23.4 (1.8, 0)
	OptiBond eXTRa Universal	18.0 (4.8, 0)	0.9 (0.3, 6)
Super-Bond Universal	Tokuyama Universal Bond II (A + B)	40.7 (4.3, 0)	23.8 (2.6, 0)
	M&C Primer A & B	31.7 (4.1, 0)	22.5 (5.2, 0)

TC 20k: after 20,000 thermocycles, n = 10, Adh: Number of adhesive failure modes after failure [21].

## Data Availability

The data presented in this study are available from the corresponding author, M.I., upon reasonable request.
